# The Actin Network Interfacing Diverse Integrin-Mediated Adhesions

**DOI:** 10.3390/biom13020294

**Published:** 2023-02-04

**Authors:** Benjamin Geiger, Rajaa Boujemaa-Paterski, Sabina E. Winograd-Katz, Jubina Balan Venghateri, Wen-Lu Chung, Ohad Medalia

**Affiliations:** 1 Department of Immunology and Regenerative Biology, Weizmann Institute of Science, Rehovot 7610001, Israel; 2 Department of Biochemistry, University of Zurich, Winterthurerstrasse 190, 8057 Zurich, Switzerland

**Keywords:** focal adhesions, integrins, cell–matrix adhesions, vinculin, actin, podosomes, invadopodia

## Abstract

The interface between the cellular actin network and diverse forms of integrin-mediated cell adhesions displays a unique capacity to serve as accurate chemical and mechanical sensors of the cell’s microenvironment. Focal adhesion-like structures of diverse cell types, podosomes in osteoclasts, and invadopodia of invading cancer cells display distinct morphologies and apparent functions. Yet, all three share a similar composition and mode of coupling between a protrusive structure (the lamellipodium, the core actin bundle of the podosome, and the invadopodia protrusion, respectively), and a nearby adhesion site. Cytoskeletal or external forces, applied to the adhesion sites, trigger a cascade of unfolding and activation of key adhesome components (e.g., talin, vinculin, integrin), which in turn, trigger the assembly of adhesion sites and generation of adhesion-mediated signals that affect cell behavior and fate. The structural and molecular mechanisms underlying the dynamic crosstalk between the actin cytoskeleton and the adhesome network are discussed.

## 1. Molecular, Structural, and Functional Diversity of Integrin-Mediated Adhesions

Integrin-mediated cell–matrix adhesions are a widespread family of membrane-cytoskeleton multi-protein complexes that play essential roles in the regulation of cell dynamics, behavior, and fate [1,2]. Members of the integrin family were shown to play key roles in multiple, often conflicting biological processes, such as tissue formation and coherence [3] and, at the same time, drive tissue degradation and remodeling [3,4]. Different forms of integrin adhesions (focal adhesions and their in vivo equivalents) provide robust interactions with the underlying matrix, which attenuate cell migration. Other forms of adhesion (such as focal complexes) provide traction forces that are needed for cell locomotion [5] and still, other forms of integrin adhesions, such as podosomes and invadopodia, play an active role in bone degradation and remodeling [6] or invasive migration of cancer cells [7].

**Figure 1:**
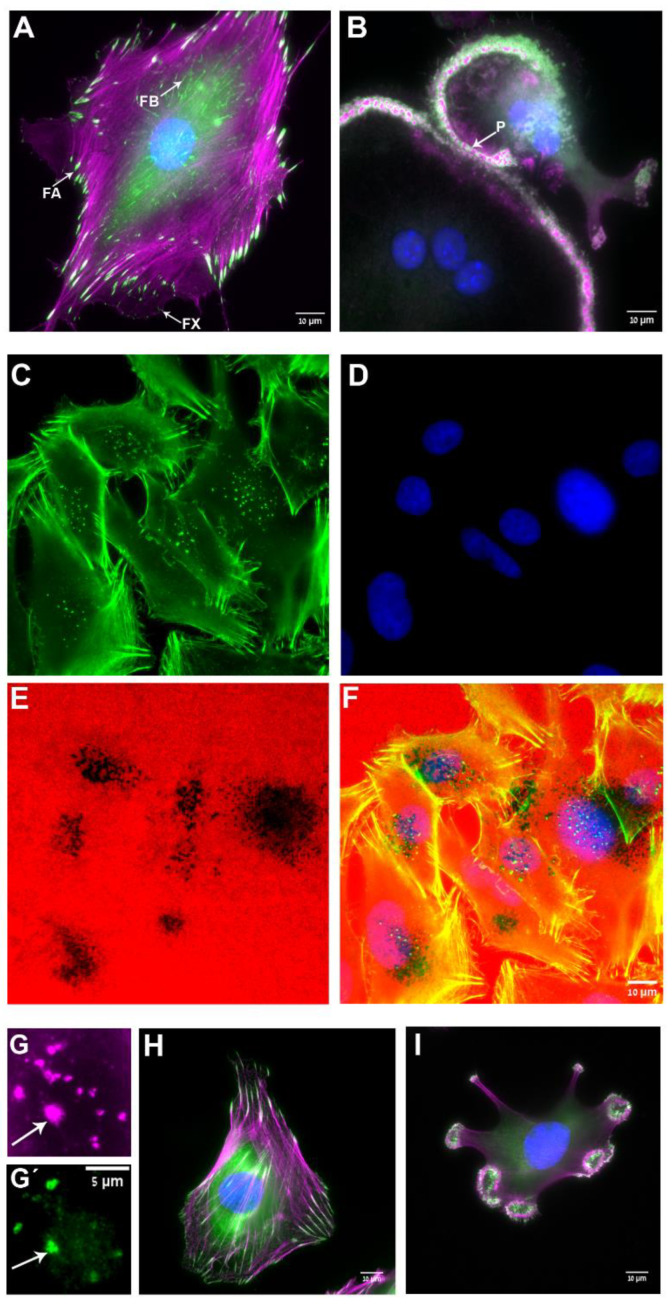
Association of F-actin with the main forms of integrin-mediated adhesions. (**A**) Vinculin-rich focal adhesions (FA, green), associated with the termini of actin-based stress fibers (magenta) in REF52 cells. Small ’focal complexes’ (FX) the precursors of focal adhesions are seen along the cell’s edge, and elongated ‘fibrillar adhesions’ (FB), associated with the ventral cell membrane, are found in the perinuclear region. (**B**) Podosomes (P) consisting of an F-actin core (magenta), and vinculin-rich adhesion ring (green) in developing (upper-right) and mature (lower-left) murine osteoclast; (**C**–**F**) cultured melanoma cell line (WM793) seeded on fluorescently labeled gelatin matrix ((**E**), red) and incubated for 5 h. The cells were then fixed and stained for actin ((**C**), green) and DAPI ((**D**), blue) for the detection of invadopodia and nuclei, respectively. The merged image is shown in (**F**). Note the prominence of actin-rich invadopodia “under” the nuclei and their close association with the dark spots, corresponding to degraded regions in the underlying gelatin matrix. (**G**) A magnified image of a WM793 cell depicting the relative distributions of actin-rich invadopodia in magenta (**G**) and vinculin in green (**G’**). The arrows point to a close association (yet, often with variable intensities) between the cytoskeletal-protrusive and the adhesive domains of the invadopodia. (**H**,**I**) Untreated MEF and MEF expressing constitutively active pp60Src (Y527F) were stained for vinculin (green) and actin (magenta). Notice the apparent transition of the adhesive cytoskeletal complex from the typical focal adhesion–stress fiber phenotype in the untreated cell (**H**) to podosome- or invadopodia-like phenotype in the cells expressing the deregulated pp60Src (**I**).

**Figure 2:**
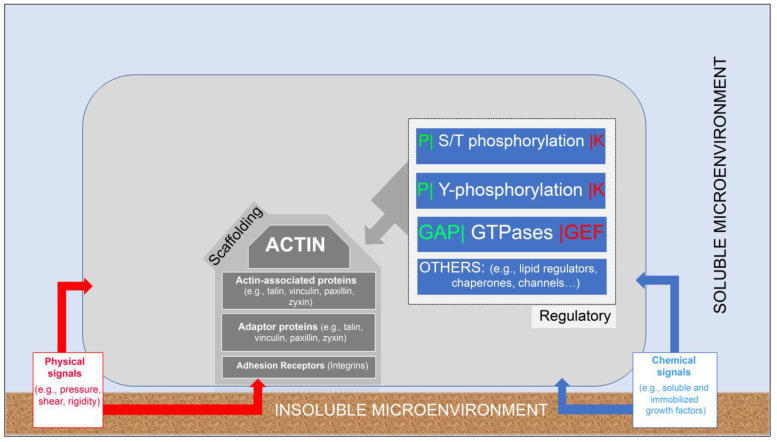
Molecular and functional networking. An illustration depicting the basic interplay between cells and the surrounding soluble and insoluble microenvironments. The integrin adhesome consists of multiple molecular components that concertedly form a “scaffolding domain” that physically links the actin cytoskeleton to the extracellular matrix and “regulatory domain” that drives the assembly and reorganization of the scaffolding components. The outermost components of the scaffolding domain are diverse heterodimeric integrin receptors that bind directly to the external surfaces. Via their transmembrane cytoplasmic “tails”, integrins interact with diverse adaptor proteins (e.g., talin, vinculin, and paxillin) that directly and indirectly connect the adhesion complex to the actin cytoskeleton. The regulatory domain consists of different signaling pathways, involving different serine and threonine (S/T)- and tyrosine (Y)-specific kinases (K) and phosphatases (P). The assembly and remodeling of the adhesion sites are affected by external signals, some of which are chemical (e.g., matrix composition, adhesive ligands, and soluble and immobilized growth factors) and others are physical (e.g., matrix rigidity, pressure, shear stress, cytoskeletal polymerization, and contraction mechanics). For further details, see “Integrin adhesions as chemical and mechanical sensors of the pericellular environment” below.

The differential, cell type-specific assembly and reorganization of these adhesions, which can drive distinct cellular processes, appears to be of great physiological significance, based on ample evidence indicating developmental or experimental deregulation of integrin adhesions can lead to severe pathological conditions, including diverse tissue deformations and immune disorders [8,9,10,11,12,13,14,15]. As shown below, some of the adhesions mentioned above (e.g., focal adhesions) can undergo pathological transformation into another form, such as invasive invadopodia, commonly present in cancer cells, where they facilitate cancer invasion [16,17,18]. Examples of these three “prototypic forms” of integrin adhesions at the light microscopy level are provided in Figure 1, which demonstrates the prominence of F-actin in and around each of these adhesions and, at the same time, their distinct overall morphologies and functions (e.g., matrix attachment, bone resorption, and cell invasion). Focal adhesions in cultured cells and their in vivo homologs (dense plaques of smooth muscle [19,20] or “sliding adhesions” to basement membranes [21]) are robust matrix adhesions that are associated with the termini of contractile actin bundles, namely stress fibers (Figure 1A). They are commonly initiated at the cell periphery in the form of focal complexes (or nascent adhesions) and are often transformed into fibrillar adhesions that are located at the cell center (Figure 1). Podosomes, prominently formed in bone-resorbing osteoclasts, are spot-like adhesions that participate in sealing the gap between the ventral membrane and the bone surface, secrete protons, and proteases into the gap, thereby supporting the resorption of the underlying bone (Figure 1B). Invadopodia, usually formed by invasive cancer cells, resemble podosomes, yet they tend to accumulate under the cells’ nuclei, and they effectively digest the underlying matrix (Figure 1C–F). Apparently, the mode of assembly and reorganization on integrin adhesions are regulated, to a large extent, at the post-translational level, mainly by a concerted activity of tyrosine-specific protein kinases, such as focal adhesion kinase (FAK) and pp60Src. Additional phosphorylation of adhesion-associated adaptors and actin-associated proteins can exert conspicuous effects on the adhesion-associated cytoskeleton and, consequently on the integrin adhesions themselves (see Figure 2). An example of a transition between adhesion forms driven by deregulated pp60Src [22] is shown in (Figure 1H) depicting mouse embryonic fibroblasts expressing constitutively active Src (Y527F) (Figure 1I). As shown, these cells lose their actin stress fibers and focal adhesions and, develop podosome- or invadopodia-like structures. Upon addition of specific Src inhibitors, the “invasive-like” phenotype is reversed in less than 2 h [23], and the stress fibers and, associated focal adhesions are restored. Taken together, these and further studies addressing the dynamic interplay between different forms of integrin adhesions point to multiple modes of regulation of their assembly or inter-conversion that, most likely, involve mechanical stimulation, activation of various signaling pathways, and different cell type-specific factors.

Interestingly, despite the apparent structural and functional diversity of these integrin adhesions, their molecular composition and overall molecular architecture bear intriguing similarities. The “integrin adhesome”, described originally in 2007 [24] and further refined over the years [25,26,27,28,29,30], consists of multiple proteins that can be divided, largely into two major groups. The first includes “scaffolding molecules”, namely, adhesion receptors, mainly integrins, key cytoskeletal components (F-actin and associated molecules), and a wide variety of adaptor proteins that bridge between the membrane-associated and the cytoskeletal domains (Figure 2, modified from [24]). The second group consists of multiple “regulatory proteins”, primarily tyrosine-specific and serine/threonine-specific protein kinases and phosphatases, GTPases, their regulators, and more (for details, see [24]). As will be discussed below, external or intrinsic cues mediated by these regulators can modulate the molecular networking of the adhesion sites.

Naturally, the integrin adhesome is a compiled list of many components based on multiple studies using different cell types and experimental approaches and, thus does not necessarily represent the actual composition of individual adhesion sites in all cells. Indeed, within the components of focal adhesions, podosomes, and invadopodia, there are many common components, such as key integrins (e.g., α5β1, αvβ3), adaptor proteins (e.g., talin, vinculin), and F-actin and associated proteins, yet there are also some apparent differences that may bear some functional significance for the different adhesion types. For example, the WASP, WIP, Arp2/3 complex, and cortactin are not highly enriched in focal adhesion; while they are prominent and play a significant role in invadopodia and podosome formation, tensin is found mainly in fibrillar adhesions [31] and zyxin is recruited to mature focal adhesions but absent from their precursors, namely focal complexes [5]. Notably, a key adaptor protein, Tks5, as well as the matrix metalloproteinase MT1-MMP (MMP14), and the actin-associated proteins, N-WASP, and gelsolin, are found in both podosomes and invadopodia but are not localized to focal adhesions [17]. Further differences in the composition, shape, and sub-cellular distribution of integrin adhesions were shown to be affected by the particular integrin heterodimer, the composition of the underlying matrix, and the internal or external mechanical forces applied to the adhesion site (see below).

A closer look into the molecular architecture of integrin adhesions and their interaction with the actin cytoskeleton was enabled by the recent advances in electron microscopy and, in particular, by the advent of cryo-electron tomography (cryo-ET) of intact eukaryotic cells [19,32,33]. This approach opened new avenues for mapping the nanoarchitecture of the cell’s actin cytoskeleton at a molecular resolution [34,35,36,37] and provided an unprecedented view of the molecular architecture of focal adhesions. Using correlative fluorescent and cryo-ET, focal adhesions were studied and the complexity of their molecular architecture was revealed. These studies suggested a laminated organization of the adhesion machinery, in which the bundled actin filaments are situated about 70 nm above the ventral plasma membrane. Adjacent to the cell membrane and, bridging the gap between the actin bundle and the membrane, doughnut-shaped macromolecular complexes were detected [34,35] whose exact molecular composition remains to be determined. Super-resolution microscopy of focal adhesions supported the laminated model and suggested that the layer between the plasma membrane and the actin filaments is filled with key scaffold-regulating adhesome proteins, such as talin, vinculin and others [30,38].

**Figure 3:**
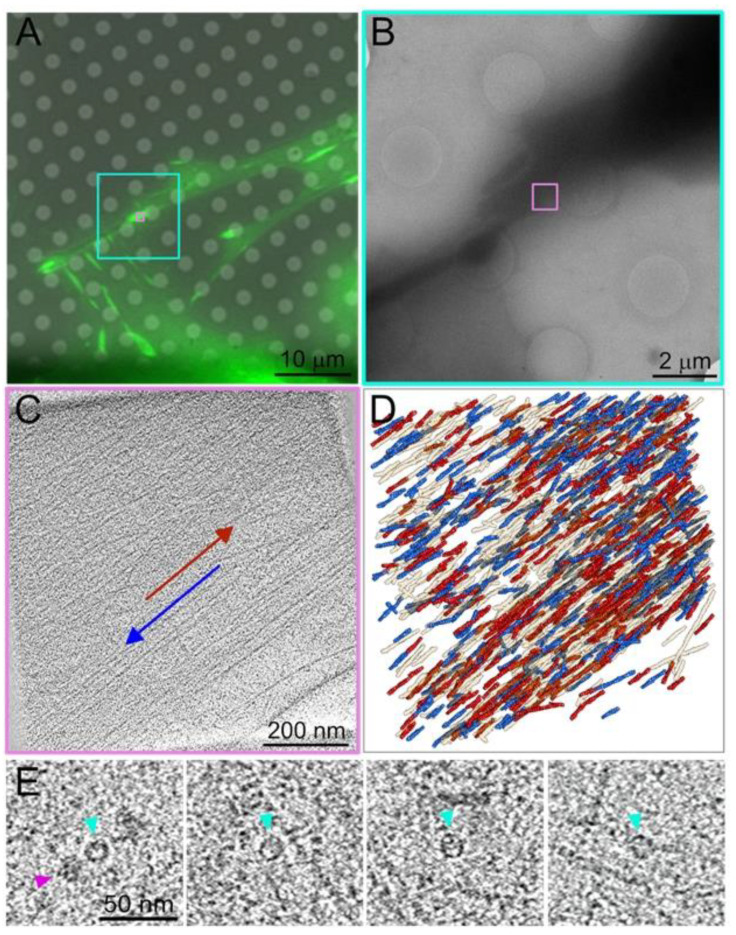
A mixed polarity of actin filaments and multiprotein complexes associated with focal adhesions. (**A**) Fluorescence microscopy image of a MEF cell expressing vinculin/venus-fluorescent protein, plated on a fibronectin-coated EM grid. The position indicated in the image, marked turquoise and magenta, was identified after vitrification using cryo-electron microscopy. (**B**) A low magnification cryo-EM image of a cell protrusion, in which the focal adhesion site was identified (turquoise) and a tomogram was acquired (Magenta). (**C**) A 9 nm thick section through a tomogram is framed in magenta (**A**,**B**). The direction of the cell edge is marked by the blue arrow and the cell interior by the red arrow. (**D**) The 3D isosurface rendering view of the tomogram is overlayed by the polarity analysis of the filaments (filaments with the barbed end toward the distal end of the cell are marked in blue, and those oriented toward the cell body are marked red (colored arrows in (**C**)). (**E**) A 9 nm slice image of the tomograms, acquired at focal adhesion, reveals macromolecular structures similar to those previously identified [34], indicated by the turquoise arrowheads. The magenta arrowheads point to ribosomes.

Recent improvements in cryo-EM enable the acquisition and analysis of the focal adhesion-associated actin and bridging particles in an unprecedented resolution that provided interesting insights into the interface between actin and the associated adhesion site. For example, the polarity of each actin filament within actin bundles or meshwork of intact and vitrified cells can now be determined in high precision, allowing us to view and quantify the filamentous actin system and its directionality. Recently, a mixed polarity of actin filaments was detected in the lamellipodia of spreading cells [39], which suggests that these F-actin networks might have the capacity to undergo myosin-driven contraction. Furthermore, actin filament mapping within the lamellopodium revealed three sub-domains, classified according to the fraction of filaments in which the barbed end points toward the plasma membrane. Surprisingly, about 10% of the actin filaments at the leading edge were found to be oriented with their barbed end toward the cell interior. Building on these technological developments, correlative light and cryo-EM studies examined the F-actin orientation around focal adhesions, revealing a conspicuous mixed polarity of actin filaments in these sites (Figure 3A–D). Whether this mixed orientation is involved in local actomyosin contractility that affects focal adhesion formation and/or maturation, as previously proposed (see [40,41]), remains to be explored in the future. Furthermore, as shown in Figure 3E, raw images of the focal adhesion nanoarchitecture, recently obtained by using cutting-edge cryo-ET technologies, enabled us to obtain much improved images of the macromolecular assemblies at’’ focal adhesion, described in our previous report [34]. At this early stage, it is difficult to predict whether extensive analysis of these raw images will be sufficient for resolving the molecular substructure of the particles or reveal the mode of their interaction with the associated actin network. It is, nevertheless, apparent that the current progress in cryo-ET technologies, the use of cryo-FIB (focused ion beam) for sample preparation, as well as emerging advanced image analysis approaches are major steps in that direction, which will enable detailed 3D molecular mapping of the nanoarchitecture not only of focal adhesions, but also invadopodia and podosomes in cultured cells and intact tissues.

## 2. A Proposed Common Feature of Integrin Adhesions: Coupling between Adhesive Domains and Nearby Protrusive Domains

**Figure 4:**
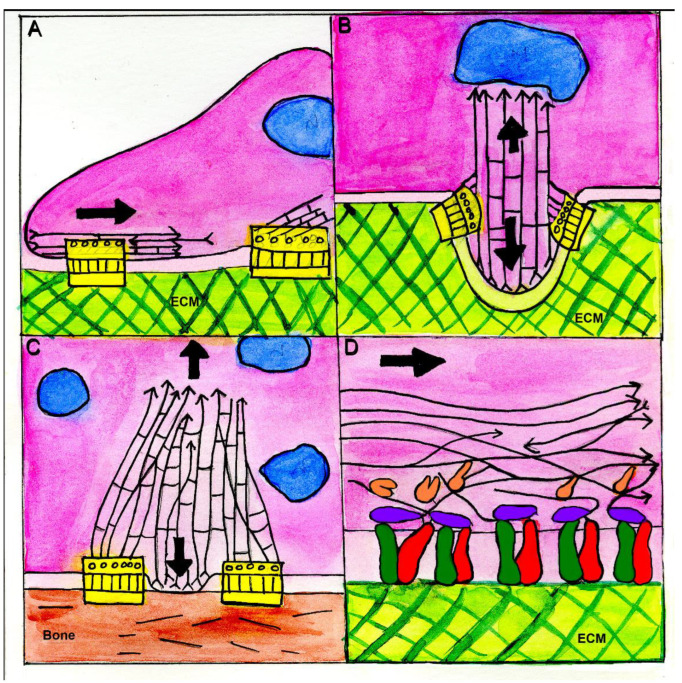
A schematic “artist’s depiction” of the protrusion–adhesion coupling model in the “classical forms” of integrin adhesions. The model shows a proposed mechanical coupling between flowing or treadmilling actin filaments and nearby integrin adhesion in four “prototypic” systems. The adhesion complexes in (**A**–**C**) are highlighted yellow, and a rough “zoom-in” into the yellow highlighted box is shown enlarged in (**D**). (**A**) Spreading of a cell (e.g., fibroblast) that forms focal complexes (left highlighted box), interacting with the centripetally flowing actin filaments in the lamellipodia, or lamellae (flow direction indicated by the thick arrow). F-actin and its polarity are represented by the black lines (arrows indicate the pointed end of the actin filaments). The right highlighted box refers to mature focal adhesions, located in more central regions of the cell. Actin-mediated forces that are applied to the nascent and mature adhesions are generated by the lamellipodial flow and the stress fiber, respectively. (**B**) A schematic view of an invadopodium, with an actin core bundle that polymerizes, thereby pushing on the ventral cell membrane and producing an invasive protrusion (down-pointing arrow). The elongating actin bundle can also apply force to the nearby nucleus (up-pointing arrow), leading to its indentation. It is proposed that in its upward treadmilling, the core actin bundle applies shear forces to the nearby adhesion site located at the “neck” of the protrusion, thereby reinforcing the adhesion. (**C**) A schematic view of a podosome located at the sealing zone of a multinuclear osteoclast, displaying a “central actin bundle” that treadmills “upwards” while pushing the ventral membrane toward the underlying bone surface, thereby contributing to the sealing of the “resorption lacuna” of the osteoclast. It is further suggested that the central actin bundle is cross-linked to “lateral actin fibers” that, in turn, interact with the nearby (ring-like) adhesion site and pull on it. (**D**) A close-up schematic depiction of the key molecular events induced by a directional flow of actin on the assembly of the integrin (colored green and red) with talin (purple) and vinculin (at different stages of unfolding; colored red). Actin filaments with different polarities are represented by arrowed black lines, with the arrow indicating the pointed end of the filaments, (for further details, see discussion of the mechanisms underlying the mechanosensing capacity of integrin adhesions below).

As pointed out above, the overall molecular composition of the different forms of integrin adhesions (focal adhesions, podosomes, and invadopodia) is quite similar, though their microarchitecture and apparent functions (namely firm adhesion to the matrix, sealing of cell-bone areas destined for resorption, and invasion into the pericellular matrix) may be quite different. We would like to consider here a possible common structural feature, which is apparently present in these three adhesion sites, namely a close interplay between an adhesive domain, made of similar sets of adhesome molecules, and a nearby protrusive domain, driven by actin polymerization. For example, focal adhesions and their focal complex precursors assemble “under” the lamellipodium where massive actin polymerization occurs, mostly at or near the leading edge, creating a rather fast (~1–3 µm/min) centripetal flow of the F-actin network [42,43,44]. When flowing over newly formed focal complexes, shear forces are applied to these nascent adhesions, which apparently stimulate their growth and the recruitment and activation of different adhesome components, which further augment the apparent friction between the flowing actin and the developing adhesion sites (see [45] and further discussion below on the effect of this flow on talin and vinculin activation). This process, in turn, triggers focal adhesion assembly, slows down the actin flow rate, and upregulates the forward extension of the leading edge [43,44,46]. This general notion of force-dependent activation of focal adhesion formation is supported by a large body of biochemical and cellular evidence for the mechanosensitivity of focal adhesions [47,48,49] and supports the view that developing focal adhesions and the nearby lamellipodium act, in fact, as mechanically and functionally coupled systems [50,51,52]. Interestingly, a similar coupling mechanism (though with a different organization and geometry) exists in invadopodia and podosomes. As suggested by the illustration in Figure 4, a directional actin flow over the cytoplasmic aspects of integrin adhesions exists not only in the developing focal adhesions, in which the streaming of the actin network comes from the lamellipodium and acts as the primary driver of both cell adhesion and cell migration (Figure 4A), but also in invadopodia, where actin polymerization in the invasive protrusion drives the mechanical penetration into the pericellular matrix and may also interact with the ring-like adhesion domain, located at the “neck” of the protrusion (Figure 4B). Another, rather unique mechanical element present in invadopodia is the apparent interaction of the elongating core actin bundle with the nucleus, which indents the nucleus and thereby enhances the pressure applied by the invasive protrusion to the underlying matrix [7]. In podosomes (Figure 4C), the actin-rich core bundle was shown to push against the ventral cell membrane, thereby potentially enhancing the sealing efficiency of the sealing zone in bone-resorbing osteoclasts [31,53,54]. This apparent centripetal treadmilling of the core actin bundle most likely pulls on the “lateral” actin filaments that are anchored to the adhesion ring [55,56]. We propose here that while the three adhesion types display an overall distinct morphology, geometry, and effect on the extracellular matrix, the interplay between flowing actin and the adhesive machinery of all three integrin adhesions is rather similar, as highlighted in Figure 4D. Direct validation of the mechanosensitive protrusion–adhesion model presented here will, most likely, require a combination of structural analysis, and fine mechanical perturbation approaches that might directly demonstrate the molecular reorganization of the adhesion sites, induced by locally-applied force. Additional aspects of the proposed protrusion–adhesion coupling are discussed in [52].

## 3. Integrin Adhesions as Chemical and Mechanical Sensors of the Pericellular Environment

**Figure 5:**
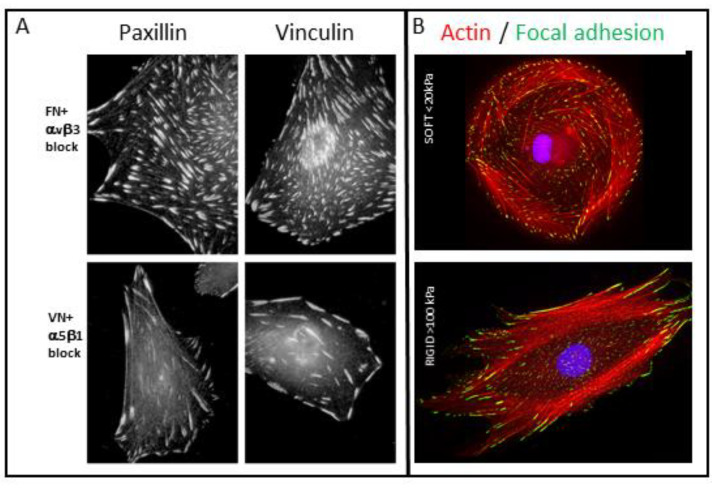
The effect of matrix composition and mechanical properties on focal adhesion and actin network organization. (**A**) Human foreskin fibroblasts (HFF) were plated in serum-free medium on glass coverslips coated with fibronectin or vitronectin and integrin-specific blocking antibodies, and were added as described. The cells were allowed to spread for 3 h, and then fixed and immunostained for vinculin or paxillin. Notice the extensive spreading of fibronectin, mediated by integrin α5β1, and broad distribution of focal adhesion throughout the entire ventral membrane compared to the reduced spreading and preferential formation of peripheral focal adhesions by cells adhering to vitronectin via integrin αvβ3. Modified from the Ph.D. thesis of B. Zimerman [70]. (**B**) HFF cells stably expressing paxillin-YFP (green) were plated on fibronectin-coated PDMS substrates with different rigidities, and fixed at 6 h following seeding. Cells were stained with TRITC–phalloidin (red) and DAPI (blue). (See also Ref. [65]). Notice the marked polarization of the cell plated on the rigid elastomer compared to the largely radial spreading on the softer matrix.

The mechanical interplay between actin-based protrusive activities and integrin adhesions is not limited to processes whereby actin-based protrusions modify the cell’s microenvironment, but it is also relevant to the capacity of integrin adhesions to act as cellular mechanosensors. In fact, integrin adhesions with the extracellular matrix were shown over the last several decades to act as sophisticated “environmental sensors” with a capacity to specifically respond to external cues, both chemical and physical. The molecular diversity of the extracellular matrix (often referred to as the “matrisome”, see [57]) is very high, with a “core matrisome” of ~300 proteins, and multiple associated growth factors and other macromolecules that concertedly affect the cell adhesion process and, eventually, the fate of the adhering cells [58,59,60]. These external matrices can be recognized specifically by diverse membrane receptors, among them several integrin α and β chains heterodimers [61], whose interaction with F-actin is the core subject of this article. The different integrins (~24 different heterodimers) can selectively recognize different matrix molecules such as fibronectin, vitronectin, collagens, laminin, fibrinogen, and thrombospondin, as well as several membrane-bound molecules [62]. Apparently, the chemical nature of the matrix and the corresponding receptors (including different integrin heterodimers) can affect the shape, cellular distribution, and affinity of the specific adhesion sites. An illustration of the differential organization of focal adhesions, mediated by different heterodimers, is shown in Figure 5A, which demonstrates that cell adhesion to fibronectin, mediated via α5β1 integrin (while blocking an alternative αvβ3 integrin) leads to extensive cell spreading with a broad distribution of paxillin- and vinculin-rich focal adhesions throughout the entire ventral aspect of the cells, while adhesion to vitronectin via αvβ3 integrin (while blocking an alternative α5β1 receptor) leads to limited cell spreading and the assembly of fewer focal adhesions with a predominant peripheral location (Figure 5A). Recently, we also demonstrated that surfaces coated with a mixture of adhesion molecules, such as fibronectin and galectin-8 (the first being a classical integrin ligand and the second driving, primarily, integrin-independent adhesion), could exert a variety of negatively- and positively-cooperative effects of cell spreading, focal adhesion formation, cell polarization and reorganization of the actin cytoskeleton, depending on their relative prominence on the external surface [63].

The physical properties of the matrix (e.g., its rigidity) can also exert dramatic effects on the mode of focal adhesion formation, cell polarization, and cytoskeletal organization [64,65,66]. As demonstrated in Figure 5B, fibroblasts spreading on a relatively rigid matrix (>100 kPa) tend to polarize and develop stress fibers that run along the major cell axis and are anchored in large focal adhesions, while cells growing on a relatively soft matrix (<20 kPa) remain largely circular with mostly radial actin bundles and smaller focal adhesions. These differences are largely attributed to the lower traction forces applied to the adhesion sites formed on soft surfaces. These and other aspects of the mechanosensing properties of integrin adhesions were described in multiple studies carried over more than two decades, demonstrating that the typical stress applied to focal adhesions (mainly by the attached stress fibers) is in the order of 5 nN/µm^2^ [31,67]. It was further demonstrated around the same time that external pulling on focal adhesions induced a remarkable growth of the adhesion site, even in the presence of actomyosin contraction inhibitors [68]. These results were consistent with earlier reports, e.g., Reference [69], indicating that treatment of cultured cells with actomyosin contractility inhibitors leads to massive disruption of focal adhesions.

The mechanosensitivity of focal adhesions, including external perturbations or internal changes in actin dynamics and contractility, and their involvement in cell behavior and cytoskeletal organization, attracted much interest within the biological and biophysical communities [71,72], leading to the emergence of “mechanobiology” as a new field in the life sciences. Some of the molecular mechanisms underlying the mechanosensitive properties of integrin adhesions will be further discussed below.

## 4. The Molecular Mechanism Underlying Actin Recruitment to Focal Adhesions: 1 The Integrin →Talin → Vinculin Connection

A large body of evidence has accumulated recently on the mechanism underlying the regulation of integrin-signaling and focal adhesion formation, focusing primarily on integrin activation and roles of two key cytoplasmic adaptor proteins, talin, and vinculin. In short, talin, in solution, exists as a folded, auto-inhibited molecule due to the binding of its “FERM” (head) domain (via its F3 sub-domain) to its Rod Segment 9 (R9) located in its tail domain. Recruitment of the folded molecule to the membrane, mediated by the interaction of its F0 subdomain with the membrane-associated GTPase Rap1 and further interaction of nearby PIP2 with its F2 and F3 sub-domains, induce conformational changes that reveal the integrin-binding site on F3, followed by partial unfolding and binding to the β1 integrin chain, which also leads to integrin activation.

The unfolding of talin leads to the exposure of multiple critical binding sites along the rod domain, enabling interaction with additional adhesome partners, including actin, paxillin, and vinculin. Further assembly processes, including the interaction of vinculin with the vinculin binding site 1 (VBS1) domain, were shown to be affected by mechanical stress [73].

Vinculin, just like talin, exists in solution, in a folded, auto-inhibited state, with its N-terminal head domain attached via its D1 and D4 subdomains to its tail [74]. Notably, most of the vinculin binding sites for different adhesome partners, including actin, talin, α-actinin, vinexin, ponsin, and paxillin [15]; Refs. [75,76,77,78,79] are not available for interaction in the folded conformation [80]. For a recent comprehensive review of the talin-vinculin-actin-integrin interplay, see [73].

A useful tool for studying molecular mechanics that mediate vinculin’s transition from the closed to the open state is a constitutively active vinculin mutant form, termed vinculin-T12, in which the vinculin tail was mutated at the region facing the D4 head domain, thereby weakening the head-to-tail interaction affinity by 100-fold [81]. Further investigation revealed that the presence of vinculin-T12 leads to an extended lifespan of multiple adhesion components in focal adhesions [82,83,84], as well as enhanced cell spreading, surface traction, and adhesion strength [85]. Surprisingly, we showed that the crystal structure obtained for this variant was of a closed conformation, nearly identical to that of the native vinculin [84]. This notion was also supported by ion mobility–mass spectrometry that indicated that vinculin and its active mutant, T12, display similar charge states and collision cross-section values. To explore the differential mechanical stability of the head–tail interaction in wild-type vinculin and the T12 mutant, we exposed these (and a few other) forms to a collision-induced unfolding perturbation, followed by ion mobility measurements. These studies indicated that the unfolding process in both forms occurred in two main steps, initially a transition from a “closed” to a “semi-open” state and, under higher collision voltage to an “open state”. That said, the collision voltage needed for the initial unfolding process was considerably lower for T12 than for the wild- type molecule [84]. Interestingly, replacing one of the four mutations in T12 with a mutation that introduces a basic amino acid (Lysine) instead of an Alanine in position 974, increased the collision voltage needed for initiating the unfolding process, but then displayed a considerably lower threshold for the transition from the semi-open to the open state. Taken together, these results suggest that an external perturbation (most likely mechanical) is needed for an effective exposure of the different binding sites, including the F-actin binding site located at the vinculin tail. It is noteworthy that the interaction between vinculin and the VBS on talin is highly mechanosensitive, so that under low forces, the VBS remains unexposed; intermediate forces expose the site and make it available for vinculin binding and high forces further lead to additional changes in the mode on the interaction between the two molecules [86].

## 5. The Molecular Mechanism Underlying Actin Recruitment to Focal Adhesions: 2 The [Integrin-Talin-Vinculin] → F-Actin Connection

**Figure 6:**
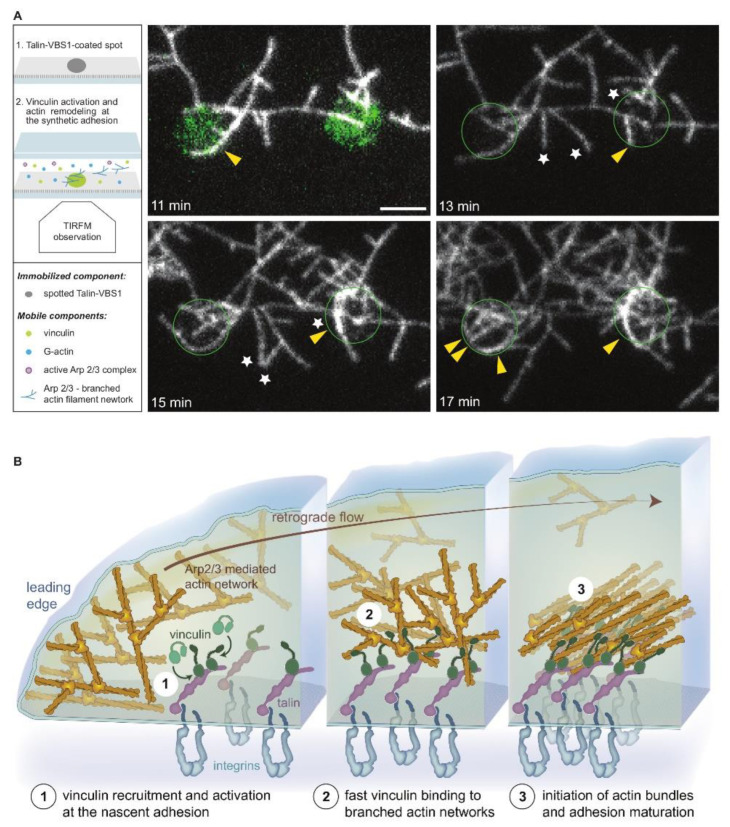
Activated vinculin remodels branched actin network into a bundle. (**A**) Activated vinculin interacts with surface-attached talin VBS1 to initiate actin bundles. A talin-VBS1 was patterned onto a glass. The patterned surface was mounted into a flow chamber, allowing a mix of inactive vinculin, actin monomers, Arp2/3, and WAVE WA (2). Bundling of Arp2/3-branched actin networks by vinculin was observed only at the sites of patterned VBS1. Arrowheads indicate bundles formed by the recruitment of actin branches labeled with stars. The circle shows the position of the VBS1-coated pattern decorated by vinculin (green in the upper left panel). Scale bar, 3 µm. (**B**) A model describing the bundling of branched actin networks by membrane-bound talin-activated vinculin at nascent adhesions. A schematic view of a nascent integrin-based adhesion site, localized in proximity to the leading edge of a cell (left). (1) The Arp2/3 branched actin network treadmills and flows centripetally. Vinculin is activated by interacting with talin [89,90]. (2) At nascent adhesion sites, talin-activated vinculin stably binds and bundles mobile branched (this work) networks, likely affecting actin dynamics [87]. (3) Actin retrograde flow generates tension that further activates talin and vinculin, reinforcing the link to integrins. Adhesion-anchored vinculin interacting with the flowing and branched actin networks initiate bundles at the nascent adhesion site. Vinculin engages the retrograde actin flow, applying tension to the mechanoresponsive components of the adhesion, enabling adhesion self-sustained assembly dynamics and the recruitment of additional focal adhesion components such as myosin II, α-actinin, and zyxin [79,87,92]. The figure was modified from [45].

Live cell video microscopy indicates that nascent integrin adhesions are formed underneath the lamellipodial branched actin network [52]. Moreover, there is compelling evidence that nascent adhesion maturation to fully-developed focal adhesions depends on the centripetal flow of lamellipodial actin, which was discussed above and depicted in Figure 4 [87]. In vitro reconstitutions assays allowed us to better define the molecular mechanisms underlying the recruitment of branched actin to the nascent adhesions. Using a constitutively active vinculin-binding site, talin (VBS1), it was shown that soluble talin-activated vinculin binds and reorganizes Arp2/3-mediated branched networks into mixed-polarity bundles similar to those found at the cell leading edge [45] (Figure 6A). Notably, using surface patterning, microfluidics technology, and high-resolution fluorescence microscopy, the stepwise recruitment and activation of vinculin to immobilize active talin-VBS1 have been reconstituted. Flowing branched actin network over immobilized, active talin–vinculin complexes lead to the formation of mixed-polarity bundles, presumably primordial stress fibers [45] similar to those observed in live cells [87]. A demonstration of a potential combined process whereby attenuation of actin flow by activated vinculin, associated with a growing focal adhesion, leads to a transition of actin from a branched network into a bundle, with mixed polarity shown in [45] and in Figure 6B.

Furthermore, recent live cell and in vitro studies showed that the release of talin autoinhibition unmasks vinculin-binding sites that recruit and activate vinculin, thus yielding talin–vinculin active precomplexes in the absence of actomyosin tensile forces [88,89,90,91]. Thus, early-stage nascent adhesion may form by the recruitment of activated talin-vinculin precomplexes that connect integrins to centripetally flowing branched actin, which in turn applies mild forces and further activates the adhesion assembly prior to full maturation under the actomyosin stress fiber force regime [79].

A recent study further suggests that vinculin undergoes a two-step allosteric maturation process to tighten its binding to talin [93]. The initial activation of vinculin occurs upon binding to talin in a force-independent manner, leading to the disruption of key salt bridges in vinculin that were identified by molecular dynamics and confirmed in a vinculin mutant, that apparently acts as a “VBS1-activated vinculin” when used in in vitro and live cells assays [93]. While the interaction between talin and vinculin can resist high actomyosin tensile forces applied on talin (above 20 pN) [94], vinculin recruitment to talin must occur when talin is subjected to a lower force range (5–10 pN) than previously measured [94], under physiological force range [95]. Under higher forces (10–20 pN), talin–vinculin is short-lived, as vinculin unbinding is faster than its maturation.

In focal adhesions, integrin-associated talin has rather slow dynamics and may then be subjected to a high-force regime for an extended time, as talin dissociates after up to 25 min [96] and focal adhesion maturation can last up to tenths of minutes [97,98]. Magnetic tweezer-based analysis reveals that talin unfolding dynamics in equilibrium is a slow process that last hours or even days. Thus, it allows for capturing rare and kinetically trapped states that were previously elusive [99]. Interestingly, the authors detected low-probability states in talin R3 and showed that the folding landscape of talin is modulated by force. As such, when kinetically trapped in a specific conformation for an extended period, talin R3 may be rescued by applying increasing forces, which were maintained in the physiological range. These low-probability states in talin R3 may be populated during focal adhesion assembly and maturation and during mechano-transduction in live cells.

In conclusion, we have attempted to illustrate, in this article, the complex interplay between actin filaments and the integrin adhesome network and demonstrate its unique capacity to explore the chemical properties of the cell’s microenvironment, as well as its physical–mechanical state. Considering the fine nanoarchitecture of different forms of integrin adhesions, we proposed a unifying view of distinct integrin adhesions based on coupling between actin-based protrusive domains and nearby adhesion sites, where mechanical forces are generated, transmitted, and sensed by cascades of protein folding and unfolding processes. A direct experimental validation of this model, combining novel imaging and biophysical technology, live cell recording, and molecular modeling is becoming the next challenge.
